# Sleep quality during pregnancy following assisted reproductive technology and natural conceiving: a prospective birth cohort study

**DOI:** 10.3389/fendo.2024.1497722

**Published:** 2025-01-22

**Authors:** Yidong Xie, Ruoti Peng, Li Xiao, Shangwei Li, Xiaohong Li

**Affiliations:** ^1^ Department of Obstetrics and Gynecology, West China Second University Hospital of Sichuan University, Chengdu, Sichuan, China; ^2^ Key Laboratory of Birth Defects and Related Diseases of Women and Children, Ministry of Education, Sichuan University, Chengdu, Sichuan, China; ^3^ West China School of Medicine, Sichuan University, Chengdu, Sichuan, China

**Keywords:** sleep quality, anxiety, depression, perceived stress, assisted reproductive technology, natural conceiving, pregnancy

## Abstract

**Purpose:**

To investigate the association of sleep quality during pregnancy on *in vitro* fertilization/intra-cytoplasmic sperm injection (IVF/ICSI) and natural conceiving (NC), as well as anxiety, depression, and perceived stress.

**Methods:**

This prospective cohort study includes 500 infertile pregnant women undergoing IVF/ICSI and 678 NC women in a Sichuan birth cohort. Data on sleep, anxiety, depression, and stress was collected in the first trimester (T1), second trimester (T2), and third trimester (T3) using integrated questionnaires. Sleep quality is quantified by the Pittsburgh Sleep Quality Index (PSQI) with a cut-point of 5 indicating poor sleep. The Self-rating Anxiety Scale (SAS), the Center for Epidemiologic Study of Depression scale (CES-D), and the Perceived Stress scale (PSS) were used for assessing anxiety, depression, and perceived stress symptoms. Additionally, the matched husbands are surveyed. Multivariable logistic regression models with adjustments for influencing factors were used to estimate odds ratios (ORs) and 95% confidence intervals (CIs) for the associations of sleep quality.

**Results:**

In the IVF/ICSI group, 61.1%, 55.5%, and 66.5% of participants in T1, T2, and T3 reported poorer sleep quality compared to the NC group, which had 43.2%, 37.4%, and 46.4% throughout the same trimesters. Additionally, the IVF/ICSI group exhibited higher levels of negative psychological factors as measured by the CES-D and PSS during T1 and T2, showing statistical significance in T1 (P = 0.008, P < 0.001) and T2 (P = 0.038, P < 0.001), except at T3 (P = 0.107, P = 0.253). In addition to psychological factors and IVF/ICSI treatment, poor sleep quality was also associated with advanced age. However, there was no significant difference in sleep quality between the husbands of the IVF/ICSI and NC groups.

**Conclusion:**

The study reveals that women receiving IVF/ICSI treatment are at a higher risk of experiencing sleep disturbances throughout their pregnancy compared with women with natural conception. While partners typically do not report major sleep problems, they do exhibit increased anxiety levels. These findings underscore the necessity for screening and addressing sleep issues in women pregnant through assisted IVF/ICSI treatment, to promote their well-being during this critical period.

## Introduction

Sleep is a vital physiological process essential for maintaining both physical and mental health, with individuals spending approximately one-third of their lifespan asleep. During pregnancy, ensuring adequate and restorative sleep is crucial not only for the mother’s well-being but also for the optimal development of the fetus. The National Sleep Foundation recommends that adults should aim for a sleep duration of seven to nine hours per night (1). A comprehensive review by Maniaci et al. highlights that conditions, like obstructive sleep apnea during pregnancy can affect maternal and fetal health, emphasizing the broader implications of sleep disturbances for pregnant women (2). These issues could negatively impact both maternal and fetal outcomes, potentially leading to complications such as preeclampsia, hypertension, gestational diabetes, cesarean deliveries, and excessive weight gain during pregnancy. However, specific guidelines for sleep duration, quality, and insomnia during pregnancy remained insufficient. Furthermore, sleep quality and disturbances are often linked to significant determinants of women’s health, particularly during critical life stages such as menstruation, pregnancy, and menopause. For instance, sleep disturbances are often associated with higher levels of depression and stress, as well as an increased risk of cardiovascular disease, hypertension, and diabetes (3). Furthermore, poor sleep quality and the prevalence of depression tend to be escalated throughout pregnancy (4). These issues could adversely affect both maternal and fetal outcomes, potentially leading to complications, including preeclampsia, hypertension, gestational diabetes, cesarean deliveries, and excessive weight gain during pregnancy (3, 5–7).

Animal studies have demonstrated that persistently disrupted light/dark cycles could significantly impair the preovulatory LH surge, leading to reduced fertility. In contrast, the reproductive effects of a single-phase shift on female mice could be minimized (8). Furthermore, sleep deprivation had been shown to diminish oocyte output, as transient activation of wake-promoting dopaminergic neurons negatively impacts reproductive performance (9). Sleep disorders were notably prevalent undergoing assisted reproductive technology (ART) treatments, and affected 57% of women undergoing *in vitro* fertilization/intra-cytoplasmic sperm injection(IVF/ICSI) before treatment, 43% during the stimulation phase, and 29% following embryo transfer (10). Philipsen et al. found that mean PSQI global scores before treatment were 8.1, with 91% of participants having PSQI scores > 5, indicating poor sleep quality (11). Research indicated that insufficient nocturnal sleep—specifically, less than seven hours per night with disrupted sleep patterns, is associated with reduced oocyte and embryo production. Conversely, excessively prolonged nocturnal sleep might decrease the likelihood of achieving a successful pregnancy. Additionally, the relationship between nocturnal sleep duration and ART outcomes is influenced by factors such as subjective sleep quality and maternal age (12).

Strong correlations have been established between sleep quality and both physical and mental well-being (13). The interplay between negative emotions and sleep difficulties can create a harmful cycle. Given that the high prevalence of sleep disorders among expectant mothers and their significant association with psychological symptoms, it is crucial to prioritize screening and counseling for psychological disorders in pregnant women to enhance sleep quality (4). Previous research also indicated that there are small but significant associations between stress and distress, which can further diminish the likelihood of successful pregnancies through ART (14). Moreover, the rates of anxiety and depression fluctuate depending on the stage of IVF/ICSI treatment. While many studies had examined women’s psychological health before and after IVF (15–17), few had focused on sleep quality and the psychological factors influencing sleep among IVF/ICSI patients during pregnancy. As a result, the relationship between sleep during pregnancy and positive psychological aspects remained poorly understood.

The existing body of research has largely focused on assessing sleep quality in individuals undergoing ART treatments. However, there is a notable gap in studies that investigate the impact of sleep quality among IVF/ICSI patients during pregnancy, particularly in comparison to women, who conceive naturally. In this study, we aim to gather data on sleep characteristics among women undergoing IVF/ICSI and those achieving natural conception. We hypothesize that both negative and positive psychological factors play a significant role in influencing sleep quality.

## Materials and methods

### Participants and study design

In this prospective cohort study, we recruited a convenience sample of 557 females who underwent ART treatment and 691 females who were natural conception and delivered at West China Second University Hospital of Sichuan University between January 2016 and July 2018. All couples included in the study were not involved in any type of prospective interventional trials. All protocols were approved by the Medical Science Ethics Board of West China Second University Hospital of Sichuan University.

### Data collection

First, each participant completed a questionnaire on demographic information and at least once of questionnaire. In this prospectively longitudinal study, data was collected in the first trimester (T1, before 12 weeks of gestation), second trimester (T2, between 13-28 weeks of gestation), and third trimester (T3, after 28 weeks of gestation) using integrated questionnaires. All the subjects were fully informed of the procedures, and their written informed consent and approval were obtained. Husbands for the matched couple were questioned once during pregnancy.

**Figure 1:**
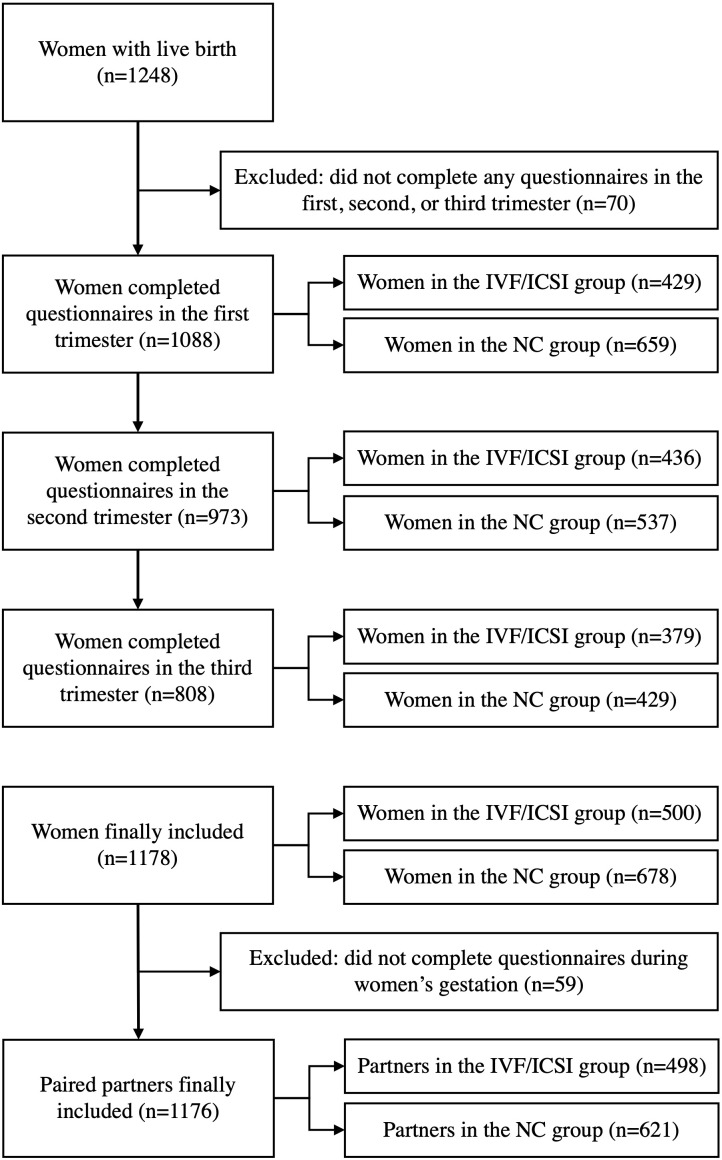
Participants flow chart.

Due to incomplete questionnaire data, 57 participants from the Assisted Reproductive Technology (ART) group and 13 from the naturally conceived (NC) group were excluded from the study as they did not respond to any questionnaires. As a result, the final analysis included 500 individuals from the IVF/ICSI group and 678 from the NC group. Simultaneously, the study encompassed 498 partners of women who conceived through IVF/ICSI and 621 partners of women who conceived naturally. A detailed inclusion and exclusion flow chart was shown in **Figure 1**.

### Pittsburgh sleep quality index

Sleep quality during pregnancy was assessed by the Pittsburgh Sleep Quality Index (PSQI), a self-reported 19-item questionnaire (18, 19). The PSQI global score, which consists of seven component scores: subjective sleep quality, subjective sleep quality, sleep latency, sleep duration, habitual sleep efficiency, sleep disturbances, use of sleeping medication, and daytime dysfunction, was used to evaluate sleep quality. Each component score was assigned a value of 0-3 points. The PSQI global score varied from 0 to 21, with higher scores suggesting worse sleep quality. In this study, sleep quality was classified into good (score ≤ 5) and poor (score> 5) according to the global PSQI scores (19). Among the poor quality, women with scores from 6 to 10 were considered fairly good, from 11 to 15 as fairly bad, and above 15 as bad sleep.

### Self-rating anxiety scale

Anxiety was measured with the Zung’s Self-Rating Anxiety Scale (SAS) (20). The scale includes 20 items that cover 4 groups of manifestations: cognitive, autonomic, motor, and central nervous system symptoms. Each item is scored on a Likert-type scale of 1-4 (1 = none or a little of the time, 2 = some of the time, 3= good part of the time, 4 = most or all of the time). Anxiety standard scores ≥ 50 were considered to be at risk for clinical anxiety. Among the anxiety group, women with scores from 50 to 60 were considered as mild anxiety, from 60 to 70 as middle anxiety, and above 70 as severe anxiety (21).

### Center for epidemiologic study of depression scale

Depression was assessed using the Center for Epidemiologic Study of Depression Scale (CES-D). The CES-D consists of 20 items which are rated using a 4-point ordered response set to indicate how frequently symptoms were experienced during the previous week (0 = rarely or none of the time, 1= some or a little of the time, 2 = occasionally or a moderate amount of the time, 3 = most or all of the time). Total score of CES-D was generated by summing their item responses and ranging from 0 to 60 (higher scores indicating more depressive symptoms). Participants with CES-D scores ≥16 were considered at risk for clinical depression (22).

### Perceived stress scale

Perceived stress was assessed with the Perceived Stress Scale (PSS-10) (23), which consists of 10 items purported to measure the degree of nonspecific appraised stress in the past month on a 5-point Likert scale (0 = never, 1 = almost never, 2= sometimes, 3 = fairly often, 4 = very often). The total PSS score ranged from 0 to 40, The stress level increases depending on the increase in score.

### Statistical analysis

SPSS 25.0 (IBM, Armonk, NY, USA) was used to analyze the study data. Baseline characteristics were summarized as mean (standard deviation, SD) for continuous variables, and frequencies (percentages) for categorical variables. Differences in these characteristics between the three nighttime sleep duration groups were compared by analysis of variance (for continuous variables) and *Chi*-square tests (for categorical variables). Nominal variables were tested either with the *Chi*-square test or Fisher’s exact test. Odds ratios (ORs) and their 95% confidence intervals (CIs) were estimated through multivariable logistic regression models to evaluate the influencing factors of the sleep quality of participants. The P-value of < 0.05 was considered statistically significant.

## Results

### Baseline characteristics and obstetric characteristics

A total of 1,178 cases of women were included in the final analysis of this study, with 500 participants in the IVF/ICSI group, with 429, 436, and 379 women in T1, T2 and T3 of pregnancy. The average( ± SD) age and pre-BMI were 32.24 ± 3.28 and 21.19 ± 2.47. Most participants were of Han ethnicity (97%). In the NC group, 678 women were included, with 659, 537, and 429 women in T1, T2 and T3 of pregnancy. The average( ± SD) age and pre-BMI were 30.67 ± 3.41 and 20.76 ± 2.78. Most participants were of Han ethnicity (99.26%). The basic characteristics of the patients are presented in **Table 1**. The rate of twin pregnancies varied significantly between the groups, with 20.6% of the IVF/ICSI-treated women and 0.88% of the NC women having twins. Furthermore, we observed that the incidence of preterm birth was 15.8% in the IVF/ICSI group compared to 4.9% in the naturally conceiving group. Meanwhile, the prevalence of intrahepatic cholestasis of pregnancy, gestational hypertension, and hypothyroidism was notably higher among women undergoing IVF/ICSI compared to those in the NC group. Additionally, the occurrence of low-birth-weight infants was significantly higher among the IVF/ICSI group than in those conceived naturally (**Table 1**).

**Table 1:** Demographic characteristic of obstetrics outcome between the IVF/ICSI and NC group.

Characteristic	IVF/ICSI group (n=500)	NC group (n=678)	P value
Female age	32.24 ± 3.28	30.67 ± 3.41	<0.001
Male age	34.10 ± 4.18	32.21 ± 4.55	<0.001
Pre-BMI, kg/m2	21.19 ± 2.47	20.76 ± 2.78	0.006
BMI at delivery	26.29 ± 2.78	26.68 ± 11.43	0.456
Weight gain during gestation	13.13 ± 4.66	13.56 ± 4.39	0.107
Education level			0.000
Middle school or less	4.60% (23/500)	5.16% (35/678)	
High school	7.00% (35/500)	2.95% (20/678)	
Graduate or higher	88.40% (442/500)	95.42% (647/678)	
Ethics			0.003
Han	97% (485/500)	99.26% (673/678)	
Minority	3% (15/500)	7.37% (5/678)	
Preconception Disease History			
PCOS			
Yes	4.2% (21/500)	1.90% (13/678)	0.023
No	5.8% (479/500)	98.10% (7/678)	
EMS			
Yes	2.8% (14/500)	1.00% (7/678)	0.027
No	97.2% (479/500)	99.00% (/678)	
Obstetrical outcomes			
Gestational Hypertension	9.40% (47/500)	3.98% (27/678)	<0.001
Gestational Diabetes Mellitus	18.80% (94/500)	16.52% (112/678)	0.301
Intrahepatic Cholestasis of Pregnancy	7.00% (35/500)	3.10% (21/678)	0.002
Placental Abruption	1.40% (7/500)	0.88% (6/678)	0.287
Placental Previa	4.00% (20/500)	1.33% (9/678)	0.237
Oligohydramnios	3.00% (15/500)	2.51% (17/678)	0.365
Intrauterine growth restriction	0.60% (3/500)	2.65% (18/678)	0.505
Macrosomia	2.00% (10/500)	2.65% (18/678)	0.301
Low birth weight infants	4.00% (20/500)	1.33% (9/678)	0.003
Multiple pregnancy	20.60% (103/500)	0.88% (6/678)	<0.001
Preterm birth	15.8% (79/500)	4.9% (33/678)	<0.001

### Sleep quality, anxiety, depression, and perceived stress in different trimesters in pregnant women

In T1, 429 women from the IVF/ICSI group, and 659 women from the NC group completed the questionnaire. The median global PSQI scores were 7 for the IVF/ICSI group and 5 for the NC group (P< 0.001). During T2, 436 women in the IVF/ICSI group participated in the survey, with537 women in the NC group. The median PSQI scores for this trimester were 6 points for the IVF/ICSI group and 5 points for the NC group (p < 0.001). In T3, 379 women from the IVF/ICSI group answered the questionnaire, compared to 429 women from the NC group. The PSQI scores were presented as a median of 7 points in the IVF/ICSI group and 5 points in the NC group (p < 0.001). Overall, when compared to the NC group during pregnancy, women undergoing ART exhibited significantly poorer sleep quality.

Pregnant women undergoing IVF/ICSI showed similar median scores on SAS throughout their pregnancy compared to those who conceived naturally (**Table 2**). Specifically, the scores were 39.79 for the IVF/ICSI group versus 39.91 for the NC group in T1 (P = 0.827), 38.52 versus 38.77 points in T2 (P = 0.874), and 40.01 versus 40.07 points in T3 (P = 0.689).

**Table 2:** Sleep quality, anxiety, depression, and stress in different trimesters between the IVF/ICSI and NC group.

Psychological Characteristics	T1		P value	T2		P value	T3		P value
	IVF/ICSI group	NC group		IVF/ICSI group	NC group		IVF/ICSI group	NC group	
	(n=429)	(n=659)		(n=436)	(n=537)		(n=379)	(n=429)	
**PSQI**	7 (4, 10)	5 (4, 7)	<0.001	6 (4, 10)	5 (3, 7)	<0.001	7 (5, 13)	5 (4, 7)	<0.001
**Sleep quality**			<0.001			<0.001			<0.001
Good	38.93%	56.75%		44.50% (194/436)	62.57% (336/537)		33.51%	53.61% (230/429)	
	(167/429)	(374/659)					(127/379)		
Fairly good	38.69%	38.39%		33.03%	33.89% (182/537)		34.04%	40.09% (172/429)	
	(166/429)	(253/659)		(144/436)			(129/379)		
Fairly bad	13.29%	4.70%		13.99% (61/436)	3.17% (17/537)		14.78%	6.29% (27/429)	
	(57/429)	(31/659)					(56/379)		
Bad	9.09%	0.15%		8.49%	0.37% (2/537)		17.68%	0.00%	
	(39/429)	(1/659)		(37/436)			(67/379)	(0/429)	
**SAS**	40 (35, 45)	40 (35, 45)	0.827	38 (33, 42)	38 (33, 43)	0.874	40 (35, 44.5)	40 (33, 45)	0.689
**Anxiety degree**			0.347			0.865			0.024
Normal	89.98%	89.38%		92.20% (402/436)	91.43% (491/537)		88.65%	87.41% (375/429)	
	(386/429)	(589/659)					(336/379)		
Mild anxiety	9.56%	9.26% (61/659)		6.65%	7.08% (38/537)		11.08%	10.02% (43/429)	
	(41/429)			(29/436)			(42/379)		
Middle anxiety	0.47%	1.37% (9/659)		1.15%	1.49%		0.26%	2.56% (11/429)	
	(2/429)			(5/436)	(8/537)		(1/379)		
**CES-D**	13 (8, 19)	15 (9, 20)	0.008	11 (6, 17)	12 (6, 19)	0.038	12 (6, 18)	13 (6, 20)	0.107
**Depressive symptoms**			0.005			0.003			0.002
No	62.94%	54.78% (361/659)		71.10% (310/436)	62.38% (335/537)		69.39%	59.67% (256/429)	
	(270/429)						(263/379)		
Yes	37.06%	45.22%		28.90%	37.62% (202/537)		30.61%	40.33% (173/429)	
	(159/429)	(298/659)		(126/436)			(116/379)		
**PSS**	13 (9, 16)	14 (11, 18)	<0.001	11 (8, 15)	13 (9, 17)	<0.001	12 (8, 16)	12 (9, 16)	0.253

T1, the first trimester; T2, the second trimester; T3, the third trimester; PSQI, Pittsburgh Sleep Quality Index; SAS, Self-Rating Anxiety Scale; CES-D, Center for Epidemiologic Study of Depression Scale; PSS, Perceived Stress Scale.

In contrast, the median scores for CES-D and PSS in the IVF/ICSI group were significantly lower than those in the NC group during T1 and T2. Specifically, the CES-D scores were 13.74 for the IVF/ICSI group compared to 15.11 for the NC group in T1 (P = 0.008) and 11.88 versus 13.1 in T2 (P = 0.038). For the PSS, the scores were 12.28 for the IVF/ICSI group versus 14.02 in T1 (P < 0.0001) and 11.51 compared to 12.56 in T2 (P < 0.001). However, in T3, the median scores of CES-D and PSS for both groups were similar across groups: CES-D was 12.63 versus 13.77 (P = 0.107) and PSS was 11.93 versus 12.29 (P = 0.253) (**Table 2**).

### The relationship between sleep quality and trimesters

**Figure 2:**
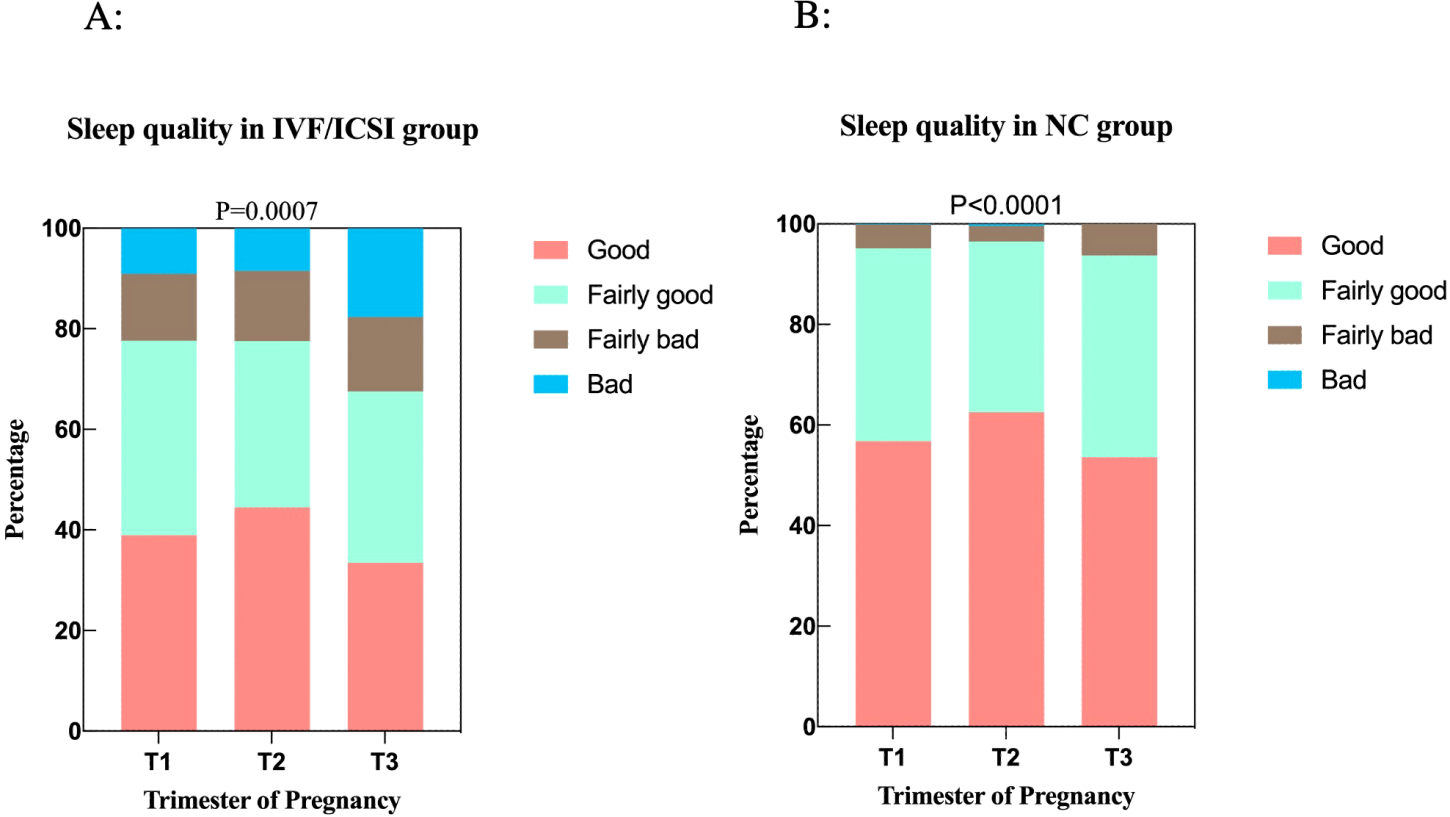
The relationship between sleep quality and trimesters. **(A)** Sleep quality in the IVF/ICSI group among three trimesters. **(B)** Sleep quality in the NC group among three trimesters.

In the IVF/ICSI group, 38.90% (167 out of 429) of women reported experiencing good sleep quality during T1, which declined to 33.5% (127 out of 379) in T3. Both of these figures were lower than the 44.5% (194 out of 436) who reported good sleep quality in T2. Similarly, in the NC group, the second trimester also showed the highest proportion of reported good sleep quality. Throughout their pregnancies, only a small number of women in the NC group reported poor sleep quality. In contrast, 17.7% (67 out of 379) of women in the IVF/ICSI group reported poor sleep quality in the third trimester, a significant increase from the 9.1% (39 out of 429) reported in T1 and 8.5% (37 out of 436) in T2. Overall, the second trimester was associated with the highest T1quality of sleep for both groups throughout the entire pregnancy (**Figure 2**).

We further studied the relationship between demographics and fertility characteristics, psychological characteristics, and sleep quality. A logistic stepwise regression model was constructed for sleep quality based on female age, pre-BMI, ethnicity, educational level, conception methods (IVF/ICSI or NC group), number of pregnancies, prior diseases (polycystic ovary syndrome and endometriosis), anxiety degree, depressive symptoms, and PPS. We found that female age, conception methods, anxiety degree, depressive symptoms, and PPS affect the sleep quality of pregnant women during the whole gestation (**Table 3**).

**Table 3:** Logistic regression analysis of sleep quality during pregnancy.

Characteristics	T1			T2			T3		
	OR	95%CI	P Value	OR	95%CI	P Value	OR	95%CI	P Value
Age (year)	0.954	0.917-0.991	0.016	0.939	0.901-0.979	0.003	0.912	0.869-0.957	<0.001
Pre-BMI (kg/m2)	0.997	0.949-1.047	0.905	1.008	0.955-1.063	0.775	1.016	0.956-1.079	0.615
Ethnicity	0.827	0.929-2.289	0.715	0.227	0.062-0.836	0.026	0.431	0.128-1.448	0.173
Educational Level	0.844	0.592-1.205	0.351	0.931	0.652-1.329	0.693	0.743	0.499-1.106	0.143
Conception method									
Natural conceiving	1			1			1		
IVF/ICSI	2.345	1.745-3.152	<0.001	2.451	1.809-3.321	<0.001	2.605	1.851-3.667	<0.001
Multiple Pregnancy	0.918	0.566-2.489	0.729	0.631	0.391-1.016	0.058	0.85	0.49-1.474	0.563
Prior PCOS	1.16	0.54-2.492	0.704	1.694	0.782-3.672	0.181	0.822	0.3-2.255	0.703
Prior EMS	0.809	0.273-2.392	0.701	0.662	0.252-1.734	0.401	0.479	0.169-1.36	0.167
Anxiety degree									
Normal	1			1			1		
Mild Anxiety	0.456	0.272-0.763	0.003	0.631	0.352-1.133	0.123	0.655	0.355-1.208	0.175
Middle anxiety	0.193	0.0023-1.584	0.126	0.125	0.016-0.999	0.05	0	0	0.999
Depressive Symptoms									
Yes	1			1			1		
No	1.891	1.402-2.55	<0.001	1.884	1.336-2.656	<0.001	1.352	0.899-2.033	0.148
PSS	0.957	0.929-0.985	0.003	0.95	0.921-0.98	0.001	0.903	0.872-0.936	<0.001

T1, the first trimester; T2, the second trimester; T3, the third trimester; BMI, Body Mass Index; PSS, Perceived Stress Scale; PCOS, Polycystic Ovary Syndrome; EMS, Endometriosis; IVF/ICSI, *In vitro* Fertilization/Intracytoplasmic Sperm Injection.

### Sleep quality, anxiety, depression, and perceived stress in paired partners

In the study, 498 matched partners from the IVF/ICSI group and 621 partners from the NC group were included. The male partners in the IVF/ICSI group exhibited slightly higher SAS scores compared to those in the NC group (P<0.001), indicating a potential increase in anxiety levels. However, there was no significant differences in sleep quality, depression levels, or stress perception of the male partners in the IVF/ICSI and NC groups (**Table 4**).

**Table 4:** Sleep quality, anxiety, depression, and stress in paired partner.

Psychological Characteristics	IVF/ICSI group (n=498)	NC group (n=621)	P value
**PSQI**	4 (2,6)	4 (2,6)	0.349
**SAS**	36 (31,40)	33 (30,38)	<0.001
**CES-D**	10 (6,10)	10 (5,16)	0.786
**PSS**	14 (11,18)	14 (11,18)	0.709

PSQI, Pittsburgh Sleep Quality Index; SAS, Self-Rating Anxiety Scale; CES-D, Center for Epidemiologic Study of Depression Scale; PSS, Perceived Stress Scale.

## Discussion

In this prospective cohort study, our findings reveal that women in the IVF/ICSI group consistently report significantly poorer sleep quality throughout all three trimesters compared to naturally conceiving women. To our knowledge, this study is the first to provide a comprehensive assessment of sleep quality, anxiety, depression, and perceived stress across different stages of pregnancy in both IVF/ICSI and naturally conceiving individuals. In the stratified analysis, we discovered that the association of sleep quality during the gestation during gestation was specifically influenced by maternal age, conception methods, anxiety status, depressive symptoms, and perceived stress. To the best of our knowledge, this is the first prospective study to evaluate sleep quality differences between the IVF/ICSI and NC groups during pregnancy.

The study revealed that a troubling trend in sleep quality among women undergoing IVF/ICSI during pregnancy. Only 38.93% reported good sleep quality in T1, decreasing to 33.51% in T3, though there was a slight improvement to 44.5% in T2. These findings are significant, as they aligned with previous research showing that pregnancy often leads to sleep disturbances, especially for those with infertility histories. Multivariate logistic regression analysis showed that age, conception methods, depression, anxiety, and perceived stress significantly impacted sleep quality. Although age was found to be significantly associated with reduced sleep quality, the group of women receiving IVF/ICSI treatment is significantly older than the natural conception group, which obviously impacts the finding of the difference between the two groups. Previous studies also have shown that sleep quality in pregnant women declines with increasing age (24). Furthermore, a meta-analysis of quantitative sleep parameters has demonstrated that sleep quality naturally diminishes as individuals age (25). Pregnant women aged 30 and older are more likely to experience stress and depressive symptoms during pregnancy, which may also elevate the risk of postpartum depression in this age group (26).

Sleep disorders are prevalent during pregnancy. Previous studies demonstrated that pregnant women experience variable levels of sleep quality deficits across all trimesters (24). PSQI scores exhibited an upward trend as pregnancy progressed, with the sleep quality of pregnant women being particularly susceptible to disruptions in the late stages of pregnancy (4). Contributing factors include emotional stress from the IVF process, physical discomfort as pregnancy progresses, and hormonal changes affecting sleep (27). Notably, the observed sleep decline in the NC group suggests that sleep issues during pregnancy are influenced by physiological and psychological demands rather than just conception methods. This study is consistent with previous findings, which suggest that around 46% of pregnant people experience sleep difficulties, with a notable drop in sleep quality occurring during the third trimester (28). This highlights the need for further investigation into sleep patterns among pregnant women, taking into account their medical histories and current health. During pregnancy, the significance of sleep for maintaining physical health was highlighted linking compromised sleep quality to negative pregnancy outcomes. The decline in sleep quality may impact maternal and fetal health, increasing risks of gestational complications, postpartum depression, and impaired maternal-fetal attachment. Studies have identified associations between sleep-disordered breathing and conditions such as gestational diabetes and hypertension (3, 29, 30). Additionally, insomnia and obstructive sleep apnea have been correlated with the increased risk of preterm birth (29).

Anxiety, depression, and stress associated with infertility treatment are essential issues. Our results reveal that while both groups exhibited similar median anxiety scores as measured by the SAS, pregnant women in the IVF/ICSI group reported significantly lower median scores on both the CES-D and PSS during T1 and T2. The retained comparability of anxiety scores across both groups throughout the pregnancy, with no statistically significant differences, suggested that the process of conception-whether through IVF/ICSI or natural means- does not lead to significant differing levels of anxiety in pregnant women. This could imply that the act of pregnancy itself, along with the accompanying physiological and emotional changes, may predispose all pregnant individuals to experience a standard level of anxiety regardless of their conception method. Significant differences in CES-D and PSS scores during the first two trimesters indicate that the IVF/ICSI group experiences lower depressive symptoms and perceived stress early in pregnancy. This may stem from a sense of accomplishment and relief from overcoming infertility. However, in the third trimester, CES-D and PSS scores equalize between groups, likely due to increased discomfortness and anxiety about childbirth affecting all pregnant women. This aligned with literature indicating that the third trimester often brings heightened physical discomfort and concerns about maternal health, impacting mental health. These findings underscored the need for individualized care approaches, suggesting that early emotional support and psychological interventions for the IVF/ICSI group could enhance their positive mental state. A study involving 842 patients undergoing IVF treatment showed that 39.4% of patients felt anxious, and 28.5% had depressive symptoms (31). Even though it is not entirely clear to what extent mental disorders affect fertility and to what extent infertility affects mental health (32), targeted interventions addressing psychological factors are crucial as pregnancies progress and challenges intensify.

The analysis of partners of pregnant women in the IVF/ICSI and NC groups revealed important insights into the emotional landscape surrounding ART and natural conception. With 498 partners from the IVF/ICSI group and 621 from the NC group, the study robustly compared mental health indicators, particularly anxiety levels measured by SAS scores. Partners in the IVF/ICSI group showed slightly higher anxiety scores, suggesting that the stress of infertility treatments affects their mental well-being. The emotional toll of IVF/ICSI, marked by uncertainty and financial strain, likely contributed to this heightened anxiety, aligning with literature on the psychological burden faced by couples undergoing fertility treatments. Interestingly, despite the increased anxiety, no significant differences in sleep quality, depression, or perceived stress were found between the two groups. This might suggest that while anxiety is a concern for IVF/ICSI partners, it does not lead to poorer sleep or higher depression levels compared to NC partners. The lack of sleep quality differences may reflect a broader trend where partners face similar sleep challenges during pregnancy, regardless of the conception method. These findings highlighted the need to consider partners’ mental health, especially for those undergoing IVF/ICSI. Future research should explore factors contributing to partner anxiety and potential interventions.

One limitation of this study is the absence of data on participants’ prior history of sleep, depression, and anxiety. This information could have provided a more comprehensive understanding of the psychological factors influencing sleep quality during pregnancy, particularly in women undergoing IVF/ICSI treatment. The lack of this data may limit our ability to fully assess the impact of pre-existing mental health conditions on the observed outcomes. Another notable limitation of this study is the reliance on self-reported questionnaires to assess sleep quality among participants. While these instruments provide valuable insights, they are subjective and may be influenced by various biases, including recall bias and social desirability bias. Previous research mainly focused on specific aspects of psychological distress without considering the interplay of multiple influencing factors. A more reliable insights into this association was highlighted. For sleep issues during pregnancy, there are pharmacological options, but there’s a lack of human data on their safety during pregnancy and lactation, raising concerns about potential birth defects and neonatal withdrawal (33). Future studies should utilize longitudinal designs to track mental health changes throughout pregnancy and identify key factors. This knowledge is vital for healthcare providers to establish support systems that help pregnant women maintain their psychological well-being during this critical period.

## Conclusions

Achieving a pregnancy and establishing a family life holds significant value for individuals undergoing IVF/ICSI as it greatly influences their overall life happiness. Consequently, even slight increases in the likelihood of achieving a pregnancy may warrant the implementation of various measures aimed at achieving success. Our study indicates that a notable concern regarding sleep quality during pregnancy among women in both IVF/ICSI and NC groups, calling for immediate attention from health professionals to address this crucial aspect of maternal health. Moreover, our current understanding of the potential impact of sleep, perceived stress, and distress on the progression from early pregnancy stage to live births remains incomplete. While IVF/ICSI partners exhibit higher anxiety, the absence of significant differences in other mental health aspects underscores the importance of understanding psychological dynamics and addressing the mental health needs of both partners to foster a supportive pregnancy environment.

## Data Availability

The original contributions presented in the study are included in the article/supplementary material. Further inquiries can be directed to the corresponding author.

## References

[1] Smiley A King D Bidulescu A. The association between sleep duration and metabolic syndrome: the NHANES 2013/2014. Nutrients. (2019) 11:2582. doi: 10.3390/nu11112582 31717770 PMC6893635

[2] Maniaci A Via LL Pecorino B Chiofalo B Scibilia G Lavalle S. Obstructive sleep Apnea in pregnancy: a comprehensive review of maternal and fetal implications. Neurol Int. (2024) 16:522. doi: 10.3390/neurolint16030039 38804478 PMC11130811

[3] Lai Y Wang C Ouyang J Wu L Wang Y Wu P. Association between nighttime sleep duration, midday napping, and sleep quality during early pregnancy and risk of gestational diabetes mellitus: a prospective cohort study in China. Sleep Med. (2024) 119:164–71. doi: 10.1016/j.sleep.2024.04.003 38685163

[4] Bahani M Zhang Y Guo Y Haretebieke S Wu D Zhang L. Influencing factors of sleep quality in pregnant: a structural equation model approach. BMC Psychol. (2024) 12:171. doi: 10.1186/s40359-024-01657-1 38528622 PMC10964610

[5] O’Brien LM Bullough AS Owusu JT Tremblay KA Brincat CA Chames MC. Pregnancy-onset habitual snoring, gestational hypertension, and preeclampsia: prospective cohort study. Am J Obstet Gynecol. (2012) 207:487.e1–9. doi: 10.1016/j.ajog.2012.08.034 PMC3505221 22999158

[6] Pauley AM Moore GA Mama SK Molenaar P Symons Downs D. Associations between prenatal sleep and psychological health: a systematic review. J Clin Sleep Med JCSM Off Publ Am Acad Sleep Med. (2020) 16:619–30. doi: 10.5664/jcsm.8248 PMC7161464 32003734

[7] Gay CL Richoux SE Beebe KR Lee KA. Sleep disruption and duration in late pregnancy is associated with excess gestational weight gain among overweight and obese women. Birth Berkeley Calif. (2017) 44:173–80. doi: 10.1111/birt.12277 28198036

[8] Bahougne T Kretz M Angelopoulou E Jeandidier N Simonneaux V. Impact of circadian disruption on female mice reproductive function. Endocrinology. (2020) 161:bqaa028. doi: 10.1210/endocr/bqaa028 32100021

[9] Potdar S Daniel DK Thomas FA Lall S Sheeba V. Sleep deprivation negatively impacts reproductive output in Drosophila melanogaster. J Exp Biol. (2018) 221:jeb174771. doi: 10.1242/jeb.174771 29361608

[10] Goldstein CA Lanham MS Smith YR O’Brien LM. Sleep in women undergoing *in vitro* fertilization: a pilot study. Sleep Med. (2017) 32:105–13. doi: 10.1016/j.sleep.2016.12.007 PMC5380145 28366321

[11] Philipsen MT Knudsen UB Zachariae R Ingerslev HJ Hvidt JEM Frederiksen Y. Sleep, psychological distress, and clinical pregnancy outcome in women and their partners undergoing *in vitro* or intracytoplasmic sperm injection fertility treatment. Sleep Health. (2022) 8:242–8. doi: 10.1016/j.sleh.2021.10.011 34949542

[12] Yao Q-Y Yuan X-Q Liu C Du Y-Y Yao Y-C Wu L-J. Associations of sleep characteristics with outcomes of IVF/ICSI treatment: a prospective cohort study. Hum Reprod. (2022) 37:1297–310. doi: 10.1093/humrep/deac040 35259255

[13] Lund HG Reider BD Whiting AB Prichard JR. Sleep patterns and predictors of disturbed sleep in a large population of college students. J Adolesc Health Off Publ Soc Adolesc Med. (2010) 46:124–32. doi: 10.1016/j.jadohealth.2009.06.016 20113918

[14] Matthiesen SMS Frederiksen Y Ingerslev HJ Zachariae R. Stress, distress and outcome of assisted reproductive technology (ART): a meta-analysis. Hum Reprod Oxf Engl. (2011) 26:2763–76. doi: 10.1093/humrep/der246 21807816

[15] Kirca N Ongen M. Perceived stress and sleep quality before oocyte pick-up, embryo transfer, and pregnancy test in women receiving *in vitro* fertilization treatment. Sleep Breath Schlaf Atm. (2021) 25:1977–85. doi: 10.1007/s11325-021-02328-w 33624218

[16] Huang L-H Kuo C-P Lu Y-C Lee M-S Lee S-H. Association of emotional distress and quality of sleep among women receiving *in-vitro* fertilization treatment. Taiwan J Obstet Gynecol. (2019) 58:168–72. doi: 10.1016/j.tjog.2018.11.031 30638474

[17] Liu Y-F Fu Z Chen S-W He X-P Fan L-Y. The analysis of anxiety and depression in different stages of *in vitro* fertilization-embryo transfer in couples in China. Neuropsychiatr Dis Treat. (2021) 17:649–57. doi: 10.2147/NDT.S287198 PMC7920591 33658786

[18] Mollayeva T Thurairajah P Burton K Mollayeva S Shapiro CM Colantonio A. The Pittsburgh sleep quality index as a screening tool for sleep dysfunction in clinical and non-clinical samples: A systematic review and meta-analysis. Sleep Med Rev. (2016) 25:52–73. doi: 10.1016/j.smrv.2015.01.009 26163057

[19] Buysse DJ Reynolds CF Monk TH Berman SR Kupfer DJ. The Pittsburgh Sleep Quality Index: a new instrument for psychiatric practice and research. Psychiatry Res. (1989) 28:193–213. doi: 10.1016/0165-1781(89)90047-4 2748771

[20] Zung WW. A rating instrument for anxiety disorders. Psychosomatics. (1971) 12:371–9. doi: 10.1016/S0033-3182(71)71479-0 5172928

[21] Zung WW Magruder-Habib K Velez R Alling W. The comorbidity of anxiety and depression in general medical patients: a longitudinal study. J Clin Psychiatry. (1990) 51 Suppl:77–80. 2189878

[22] Roberts RE Vernon SW. The Center for Epidemiologic Studies Depression Scale: its use in a community sample. Am J Psychiatry. (1983) 140:41–6. doi: 10.1176/ajp.140.1.41 6847983

[23] Cohen S Kamarck T Mermelstein R. A global measure of perceived stress. J Health Soc Behav. (1983) 24:385–96. doi: 10.2307/2136404 6668417

[24] Sedov ID Cameron EE Madigan S Tomfohr-Madsen LM. Sleep quality during pregnancy: A meta-analysis. Sleep Med Rev. (2018) 38:168–76. doi: 10.1016/j.smrv.2017.06.005 28866020

[25] Ohayon MM Carskadon MA Guilleminault C Vitiello MV. Meta-analysis of quantitative sleep parameters from childhood to old age in healthy individuals: developing normative sleep values across the human lifespan. Sleep. (2004) 27:1255–73. doi: 10.1093/sleep/27.7.1255 15586779

[26] Gao M Hu J Yang L Ding N Wei X Li L. Association of sleep quality during pregnancy with stress and depression: a prospective birth cohort study in China. BMC Pregnancy Childbirth. (2019) 19:444. doi: 10.1186/s12884-019-2583-1 31775666 PMC6882237

[27] Andersen ML Hachul H Ishikura IA Tufik S. Sleep in women: a narrative review of hormonal influences, sex differences and health implications. Front Sleep. (2023) 2:1271827. doi: 10.3389/frsle.2023.1271827 PMC12713927 41426438

[28] Pengo MF Won CH Bourjeily G. Sleep in women across the life span. Chest. (2018) 154:196–206. doi: 10.1016/j.chest.2018.04.005 29679598 PMC6045782

[29] Facco FL Parker CB Reddy UM Silver RM Koch MA Louis JM. Association between sleep-disordered breathing and hypertensive disorders of pregnancy and gestational diabetes mellitus. Obstet Gynecol. (2017) 129:31–41. doi: 10.1097/AOG.0000000000001805 27926645 PMC5512455

[30] Wilson DL Walker SP Fung AM Pell G O'Donoghue FJ Barnes M. Sleep-disordered breathing in hypertensive disorders of pregnancy: a BMI-matched study. J Sleep Res. (2018) 27:e12656. doi: 10.1111/jsr.12656 29368415

[31] Xu H Ouyang N Li R Tuo P Mai M Wang W. The effects of anxiety and depression on *in vitro* fertilisation outcomes of infertile Chinese women. Psychol Health Med. (2017) 22:37–43. doi: 10.1080/13548506.2016.1218031 27686881

[32] Szkodziak F Krzyżanowski J Szkodziak P. Psychological aspects of infertility. A systematic review. J Int Med Res. (2020) 48:300060520932403. doi: 10.1177/0300060520932403 32600086 PMC7328491

[33] Miller MA Mehta N Clark-Bilodeau C Bourjeily G. Sleep pharmacotherapy for common sleep disorders in pregnancy and lactation. Chest. (2020) 157:184–97. doi: 10.1016/j.chest.2019.09.026 PMC6965691 31622589

